# A Bibliometric Analysis of the Landscape of Problem-Based Learning Research (1981–2021)

**DOI:** 10.3389/fpsyg.2022.828390

**Published:** 2022-03-15

**Authors:** Fan Zhang, Hui Wang, Yan Bai, Huachun Zhang

**Affiliations:** ^1^Department of Nephrology, Longhua Hospital Shanghai University of Traditional Chinese Medicine, Shanghai, China; ^2^Department of Anorectal, Longhua Hospital Shanghai University of Traditional Chinese Medicine, Shanghai, China; ^3^Department of Cardiology, Longhua Hospital Shanghai University of Traditional Chinese Medicine, Shanghai, China; ^4^Department of Nursing, Longhua Hospital Shanghai University of Traditional Chinese Medicine, Shanghai, China

**Keywords:** problem-based learning, bibliometric analysis, education, citation, research

## Abstract

**Background:**

Problem-Based Learning (PBL) is an instructional method of hands-on, active learning centered on investigating and resolving messy, real-world problems. This study aims to systematically analyze the current status and hotspots of PBL research and provide insights for research in the field.

**Methods:**

Problem-based learning-related publications were retrieved from the Web of Science Core Collection using “Problem-Based Learning”. Annual publications, countries, institutions, authors, journals, references, and keywords in the field were visually analyzed using the R, VOSviewer, and Microsoft Excel 2019 software.

**Results:**

A total of 2,790 articles and reviews were analyzed, with a steady increase in publications in the field of PBL. Overall, the United States was the major contributor to the study of PBL. Van Der Vleuten CPM was the key researcher in this field. Moreover, most of the publications were published in *Medical Education*. Keyword analysis showed that current research hotspots focus on the extensions of PBL teaching mode, application of PBL teaching method, and reform of PBL.

**Conclusion:**

Research on PBL is flourishing. Cooperation and exchange between countries and institutions should be strengthened in the future. These findings will provide a better understanding of the state of PBL research and inform future research ideas.

## Introduction

Problem-based learning (PBL) is a pedagogy that has received widespread attention in recent years (Albanese and Mitchell, 1993). It emphasizes setting learning into complex problem situations, allowing learners to solve authenticity problems collaboratively, understand the scientific knowledge implicit behind the problems (Wood, 2003; Dolmans et al., 2005). In addition to course content, PBL can promote the development of critical thinking skills, problem-solving abilities, and communication skills (van der Vleuten and Schuwirth, 2019). It can also allow working in groups, finding and evaluating research materials, and life-long learning (Compton et al., 2020).

As a broad approach, PBL first originated in medical education in the 1960s at the medical school at McMaster University in Canada (Jones, 2006) and has since been promoted and modified in more than 60 medical schools (Servant-Miklos et al., 2019). PBL was most used in the first 2 years of medical courses, replacing traditional teaching methods in anatomy, pharmacology, and physiology (Devine et al., 2020). Today, PBL has been widely used in business, dentistry, health sciences, law, engineering, education (Huth et al., 2021; Kühner et al., 2021; Michalsky and Cohen, 2021).

Bibliometrics analysis refers to the qualitative and quantitative evaluation of specific research areas using mathematical and statistical methods to understand the knowledge structure and explore development trends (Bornmann and Leydesdorff, 2014). In recent years, bibliometric types of research have received extensive attention to provide a comprehensive overview of the published literature and identify research frontiers and future research trends (Liu et al., 2021; Shawahna, 2021; Yu et al., 2021).

Previously published bibliometric studies on PBL have been limited to highly cited articles (Azer, 2017). In order to understand the research trends of PBL teaching, the aim of the study, therefore, is to analyze international scientific publications using both quantitative and qualitative bibliometric analysis on PBL teaching. This work will provide new perspectives and references for future PBL research.

## Materials and Methods

### Data Sources

Publications about PBL were retrieved from the Web of Science Core Collection database. The database covers over 21,000 peer-reviewed, high-quality academic journals, including open access journals published in over 250 medical, social science and humanities disciplines worldwide, and is widely used for bibliometric analysis.

Moreover, the database provides access to the authors (country), affiliation, keywords, and references cited for each publication, which is necessary for this study.

### Search Strategy

The searched strategy was TS = “Problem-Based Learning” from inception to 27 October 2021. No language restrictions. A total of 3,339 publications was retrieved, and after excluding meeting abstracts, editors, letters, and corrections, 2,790 publications were included, of which 156 were reviews, and 2,634 were articles.

### Data Analysis

All downloaded documents were imported to the R (version 4.1.1), VOSviewer (version 1.6.15), and Microsoft Excel 2019.

Bibliometrix R package is an open-source tool for quantitative research in scientometrics and bibliometrics (Aria, 2017). VOSviewer is a software tool for constructing and visualizing bibliometric networks, including countries, journals, and authors based on citation, co-citation, or co-authorship relations. VOSviewer also offers text mining functionality that can be used to construct and visualize co-occurrence networks of important terms extracted from a body of scientific literature (van Eck and Waltman, 2010). Scientific knowledge mapping can intuitively understand the research hotspots and development process of each field in the knowledge system and predict the development trend of each field (Chen, 2004).

## Results

### Trends in Global Publication

**FIGURE 1:**
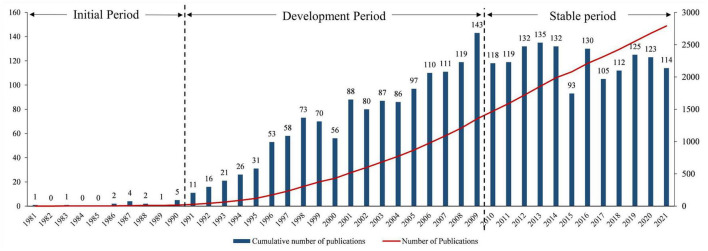
Annual number of publications in the field of PBL research.

Based on the number of annual publications, this period was preliminarily divided into three phases (Figure 1): the first phase is the initial period (1981–1990), with an average of two publications per year; the second phase, from 1991 to 2009, was considered as the development period, with an average of annual publications of 70; and the third phase, from 2010 to present, was known as the stable period when the annual number in this period was at a relatively stable state, and 120 publications were published annually.

### Distribution of Countries/Regions

**FIGURE 2:**
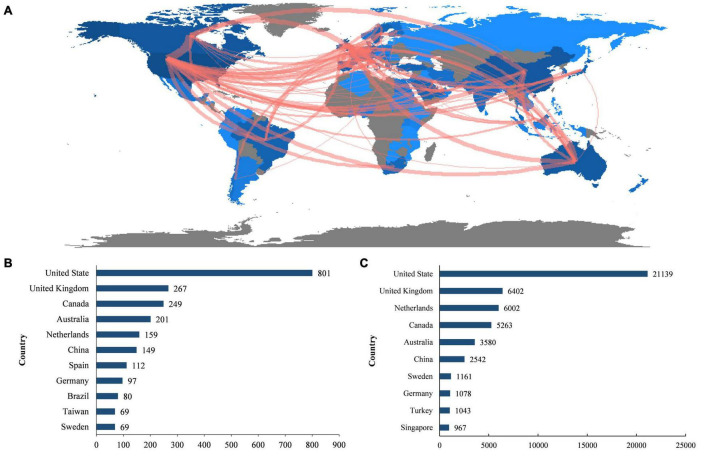
Countries were contributing to PBL research. **(A)** World map of the number of publications published by countries. **(B)** Top ten countries with the largest number of publications. **(C)** Total citations of publications from different countries.

A world map based on the number of publications published in each country is shown in Figure 2A. A total of 87 countries/regions have been published in the field. The United States contributed the most publications (801, 28.71% of all publications), followed by the United Kingdom (267, 9.57%), Canada (249, 8.92%), Australia (201, 7.20%), and the Netherlands (159, 5.70%) (Figure 2B). Publications from the United States (21,139 citations) were the most cited, with the United Kingdom (6,402 citations), the Netherlands (6,002 citations), Canada (5,263 citations), and Australia (3,580 citations) ranking second through fifth, respectively (Figure 2C).

The co-authorship analysis found a total of 56 countries/regions with at least five publications published in this field. The five countries with the highest total link strength were the United States (total link strength = 150), the United Kingdom (total link strength = 132), the Netherlands (total link strength = 93), Canada (total link strength = 79) and Australia (total link strength = 65). The network of cooperative relationships between countries is shown in Figure 2A.

### Distribution of Institutions

**FIGURE 3:**
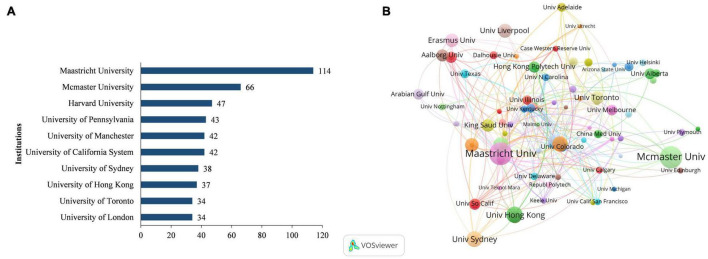
Institutions were contributing to PBL research. **(A)** Top ten institutions with the largest number of publications. **(B)** Network map of co-authorship between institutions with more than five publications.

A total of 1,973 institutions have published papers in this field. Among them, the Maastricht University contributed the most (95 records), followed by McMaster University (66 records), Harvard University (47 records), University of Pennsylvania (43 records), and University of Manchester (42 records) (Figure 3A).

We analyzed co-authorship relationships between 187 institutions with at least five publications. Excluding the 24 unconnected items, Figure 3B shows the collaborations of 163 institutions. The five institutions with the highest connection total link strength were Maastricht University (total link strength = 34), Erasmus University Rotterdam (total link strength = 25), Harvard University (total link strength = 25), the University of Sydney (total link strength = 18), and Johns Hopkins University (total link strength = 14).

### Analysis of Journals and Research Areas

There are 2,890 papers published in 608 journals. Table 1 lists the top ten most popular journals for publishing papers on PBL. *Medical Education* published 235 articles, by far the most, followed by *Medical Teacher* (*n* = 194), *International Journal of Engineering Education* (*n* = 127), *Advances in Health Sciences Education* (*n* = 111), *Academic Medicine* (*n* = 100).

**TABLE 1:** The top ten popular journals and cited journals.

Rank	Journals	Records (n)	2020 IF	Cited journals	Records (n)	2020 IF
1	Medical Education	235	6.251	Medical Education	4,757	6.251
2	Medical Teacher	194	3.650	Academic Medicine	4,482	6.893
3	International Journal of Engineering Education	127	0.969	Medical Teacher	2,252	3.650
4	Advances in Health Sciences Education	111	3.853	Journal of Dental Education	894	2.264
5	Academic Medicine	100	6.893	Advances in Health Sciences Education	817	3.853
6	BMC Medical Education	86	2.463	Nurse Education Today	773	3.442
7	Nurse Education Today	72	3.442	Problem Based Learning	732	–
8	Biochemistry and Molecular Biology Education	69	1.160	Teaching and Learning in Medicine	647	2.414
9	American Journal of Pharmaceutical Education	66	2.047	American Journal of Pharmaceutical Education	624	2.047
10	Journal of Dental Education	62	2.264	Journal of Chemical Education	589	2.979

**FIGURE 4:**
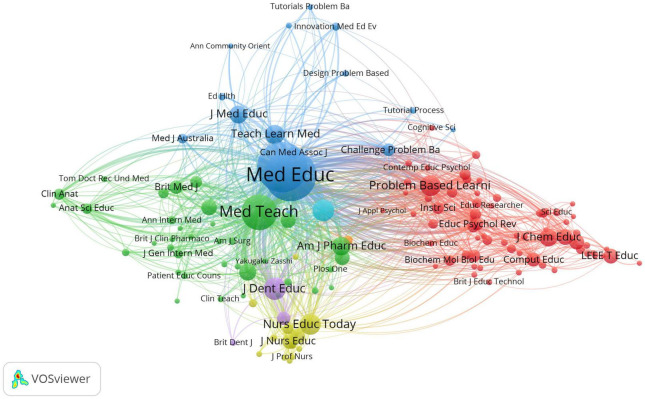
Network map of journals that were co-cited at least 50 publications.

We analyzed a total of 141 journals that were co-cited at least 50 publications (Figure 4). Table 1 lists the top ten journals. Of these, *Medical Education* has the most citations (4,757 citations), followed by *Academic Medicine* (4,482 citations), *Medical Teacher* (2,252 citations), *Journal of Dental Education* (894 citations), and *Advances in Health Sciences Education* (817 citations).

The included publications were categorized into 108 research areas. The most representative research areas were Educational Research (1,573 records), HealthCare Sciences (774 records), Engineering (359 records), General Internal Medicine (230 records), Nursing (212 records) (Table 2).

**TABLE 2:** The top ten representative research areas.

Rank	Research areas	Records (n)	% (of 2,790)
1	Educational research	1,573	56.38%
2	Healthcare sciences	774	27.74%
3	Engineering	359	12.87%
4	General internal medicine	230	8.24%
5	Nursing	212	7.60%
6	Pharmacology pharmacy	153	5.48%
7	Computer science	148	5.30%
8	Dentistry oral surgery medicine	113	4.05%
9	Biochemistry molecular biology	88	3.15%
10	Physiology	61	2.19%

### Analysis of Authors

**FIGURE 5:**
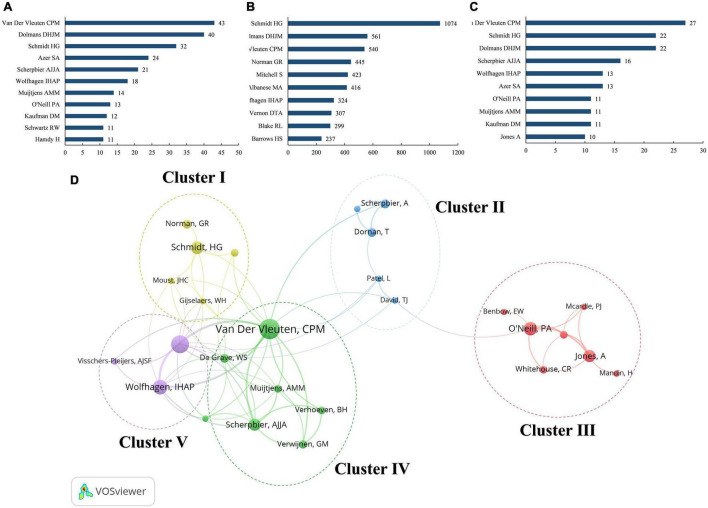
Analysis of authors. **(A)** The number of author publications. **(B)** Total citations from different authors in the field of PBL. **(C)** *h*-index for authors. **(D)** Network map of co-authorship between authors with more than three publications.

In terms of the number of publications, Van Der Vleuten CPM was the most prolific author (*n* = 43), followed by Dolmans DHJM (*n* = 40), Schmidt HG (*n* = 32), Azer SA (*n* = 24), Scherpbier AJJA (*n* = 21) (Figure 5A). From the author’s influence, Schmidt HG has the largest number of citations in this field (1,074), followed by Dolmans DHJM (561), Van Der Vleuten CPM (540), Norman GR (445), Mitchell S (423) (Figure 5B). Publications from Van Der Vleuten CPM had the highest *h*-index (27), followed by Schmidt HG (22), Dolmans DHJM (22), Scherpbier AJJA (16), Wolfhagen IHAP (13) (Figure 5C).

We further analyzed a total of 212 authors that were co-authorship in more than three publications. After removing non-connected authors from each other, the network shows the collaboration of 29 authors (Figure 5D). The five authors with the highest total link strength were Van Der Vleuten CPM (total link strength = 66 times), Dolmans DHJM (52), Wolfhagen IHAP (40), Scherpbier AJJA (33), and Schmidt HG (32).

### Citation and Co-citation Analysis

**FIGURE 6:**
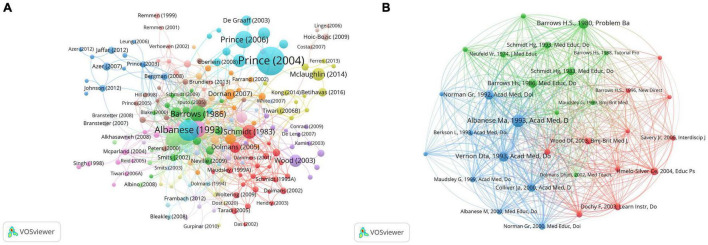
Citations analysis. **(A)** Network map of citation analysis of documents with more than 50 citations. **(B)** Network map of co-citations analysis of references with more than 50 citations.

The citation analysis showed that 243 documents had at least 50 citations (Figure 6A). Table 3 lists the top ten documents with the highest citations. In addition, we analyzed the 32 references that were co-cited in more than 50 citations (Figure 6B). Table 4 lists the top ten references with the highest citations.

**TABLE 3:** Top ten citations analysis of publications on this field.

Rank	Title	First Author	Source	Publication Year	Citations (n)
1	Problem-based learning: a review of literature on its outcomes and implementation issues (Albanese and Mitchell, 1993)	Albanese MA	Academic Medicine (IF: 6.893)	1993	413
2	Does problem-based learning work? A meta-analysis of evaluative research (Vernon and Blake, 1993)	Vernon DTA	Academic Medicine (IF: 6.893)	1993	266
3	The psychological basis of problem-based learning: a review of the evidence (Norman and Schmidt, 1992)	Norman GR	Academic Medicine (IF: 6.893)	1992	234
4	A taxonomy of problem-based learning methods (Barrows, 1986)	Barrows HS	Medical Education (IF: 6.251)	1986	198
5	Effectiveness of problem-based learning curricula: research and theory (Colliver, 2000)	Colliver JA	Academic Medicine (IF: 6.893)	2000	177
6	Problem-based learning: rationale and description (Schmidt, 1983)	Schmidt HG	Medical Education (IF: 6.251)	1983	171
7	Problem based learning (Wood, 2008)	Wood DF	BMJ (IF: 39.890)	2003	123
8	Foundations of problem-based learning: some explanatory notes (Schmidt, 1993)	Schmidt HG	Medical Education (IF: 6.251)	1993	115
9	Effectiveness of problem-based learning curricula: theory, practice and paper darts (Norman and Schmidt, 2000)	Norman GR	Medical Education (IF: 6.251)	2000	102
10	Problem-based learning: future challenges for educational practice and research (Dolmans et al., 2005)	Dolmans DHJM	Medical Education (IF: 6.251)	2005	94

**TABLE 4:** Top ten co-citation analyses of cited references on this field.

Rank	Title	First author	Source	Publication year	Citations (n)
1	Problem-based learning: a review of literature on its outcomes and implementation issues (Albanese and Mitchell, 1993)	Albanese MA	Academic Medicine (IF: 6.893)	1993	413
2	Problem-Based Learning:An Approach to Medical Education (Barrows and Tamblyn, 1980)	Barrows HS	New York: Springer	1980	270
3	Does problem-based learning work? A meta-analysis of evaluative research (Vernon and Blake, 1993)	Vernon DTA	Academic Medicine (IF: 6.893)	1993	266
4	The psychological basis of problem-based learning: a review of the evidence (Norman and Schmidt, 1992)	Norman GR	Academic Medicine (IF: 6.893)	1992	234
5	A taxonomy of problem-based learning methods (Barrows, 1986)	Barrows HS	Medical Education (IF: 6.251)	1986	198
6	Effectiveness of problem-based learning curricula: research and theory (Colliver, 2000)	Colliver JA	Academic Medicine (IF: 6.893)	2000	177
7	Problem-based learning: rationale and description (Schmidt, 1983)	Schmidt HG	Medical Education (IF: 6.251)	1983	171
8	Problem-based learning: What and how do students learn? (Hmelo-Silver, 2004)	Hmelo-Silver CE	Education Psychology Review (IF:5.167)	2004	157
9	Problem based learning (Wood, 2003)	Wood DF	BMJ (IF:30.223)	2003	123
10	Effects of problem-based learning: A meta-analysis (Dochy et al., 2003)	Dochy F	Learn and Instruction (IF:3.323)	2003	116

### Co-occurrence Analysis of Keywords

**FIGURE 7:**
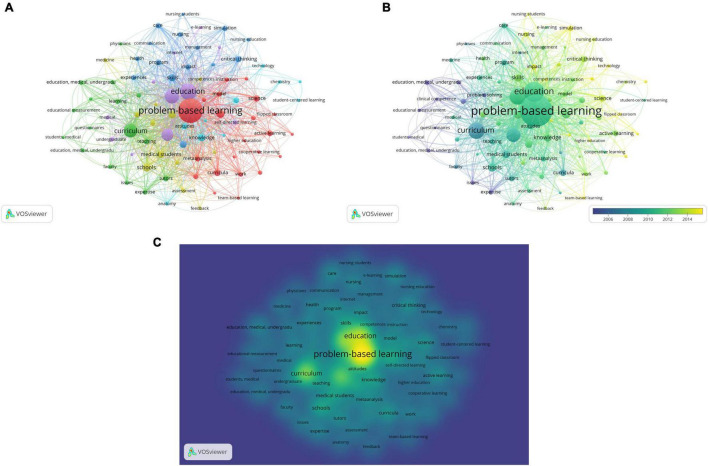
Co-occurrence analysis of keywords. **(A)** Network visualization. **(B)** Overlay visualization. **(C)** Density visualization.

We analyzed a total of 86 keywords that were identified as having occurred more than five times (Figure 7A). The colors in the overlay visualization shown in Figure 7B indicate the average publication year of the identified keywords. Most keywords were published after 2012, with greener or yellower colors. The density visualization showed the exact identified keywords mapped by frequency of appearance (Figure 7C).

## Discussion

This study analyzed the bibliometric properties of 2,790 publications included in a citation index of PBL studies conducted over the past 40 years. The trend of annual publications demonstrated that the studies during this period were stable growth. The bibliometric results provide researchers, policymakers, and teaching staff with valuable insights and enable them to get meaningful references based on objective data.

A quantitative and visual analysis of the distribution of countries/regions and institutions shows that the United States and the United Kingdom are the leading countries where PBL research is being conducted. As shown in Figure 3A, there is a greater density and breadth of collaboration between the various countries. Research teams in the United States mainly collaborated with the United Kingdom, Canada, China, Australia, and Europe. In addition, although each institution has its collaborative network, the breadth and intensity of the collaboration are not ideal. The cooperation center mainly revolves around Maastricht University, Mcmaster University, and Havard University, the three institutions with the largest publications. The intricacies of the mapping illustrate two things: first, the close cooperation among institutions that have contributed to the results of PBL research, and second, the continuous development of PBL in the teaching of different disciplines.

Problem-based learning is a problem-oriented teaching method (Savery and Duffy, 1997). It is a teaching model in which students collect information independently around problems, find out and solve problems, and develop independent learning and innovation abilities (Domingo-Osle et al., 2021). Most studies of PBL were published in influential education-related journals such as *Medical Education* and *Medical Teacher*. Regarding co-cited journals, we can see that most studies were from high-impact journals. These journals are equally focused on education and influence the direction of research in the field. As shown in Table 2, in addition to educational research, PBL teaching has now been extended to clinical medicine, engineering, computer science. This result is similar to the findings of another study, in which Azer found that highly cited articles in the field of PBL were distributed among journals in dental and medical education, general medicine, and teaching psychology (Azer, 2017).

In the past decade, the focus of teaching and learning, including medical education, has gradually shifted to developing students’ problem-solving, critical thinking, and self-directed learning skills (Merisier et al., 2018). PBL is being adopted and valued by an increasing number of universities and hospitals as a teaching model that fits well with constructivist learning theory and medical teaching principles (Al-Azri and Ratnapalan, 2014). This phenomenon is corroborated by the results of PBL posting journals and citations presented in Tables 1–4. Dentistry stands out in medical education as one of the most widely implemented disciplines for PBL teaching. Various branches of dentistry such as prosthodontics and orthodontics are convenient subjects and have close cross-fertilization with many fields such as material science, clinical medicine, pathology, physiology (Ferro et al., 2019). Therefore, dentistry teaching requires students to be proficient in dentistry-related courses and, more importantly, to apply and integrate them. As Azer said, the bibliometric analysis of PBL has implications for dental teaching and research (Azer, 2017).

The most prolific authors in PBL studies and the global citations to their work differed. The most prolific and influential author is Van Der Vleuten CPM, while the most cited author was Schmidt HG. In terms of the number of citations, “PBL: a review of literature on its outcomes and implementation issues” (Albanese and Mitchell, 1993) published by Albanese MA was the more influential article, consistent with Azer’s bibliometric results (Azer, 2017), suggesting that this article is a classic citation in the field of PBL. The study compared the effects of PBL teaching and traditional teaching through meta-analysis, thus pointing out the advantages and disadvantages of PBL teaching. However, as shown in Figure 5D, the range of co-author can be roughly divided into five clusters, and the density of collaboration between authors is lower, which may be related to the interdisciplinary application of PBL.

From keyword analysis, the current research focuses on three orientations: (1) Extensions of PBL teaching mode, such as case-based learning, flipped classroom, team-based learning. The pedagogical research around PBL gradually extends to different teaching modes, which is to promote better active and positive learning of learners and the development of education. (2) Applying PBL methods to clinical medicine, especially nursing; “Question” is the best way to promote critical thinking, which is urgently needed in modern nursing to promote the overall quality of nurses; hence, its proper use is essential in fostering the development of clinical reasoning. (3) The reform of PBL, like think, challenge, and decision making. Today, problem-oriented teaching models often involve computer-based programs. Regardless of the technique used, the core of the approach remains the same: real-world problems. Reflections on the PBL reform could be the future direction of the following research in this field.

It is worth noting that the bibliometric analysis also provides new ideas for teaching research. First, PBL is a student-centered teaching model that has been widely used in various disciplines. However, different teaching characteristics in different disciplines exist, and long-term quantitative assessments of its effectiveness are scarce. Second, Problem-based learning is an advanced teaching method, but the classical, traditional teaching methods cannot be rejected wholesale due to reform needs; both can coexist and complement each other’s strengths (Payne, 2004). From the teachers’ perspective, planning the important and difficult points of learning and developing targeted discussion outlines to motivate students is undoubtedly the key to PBL research. Third, questions are the core of PBL, and all learning activities revolve around questions. However, the purpose of PBL is to accomplish the course objectives, such as developing students’ knowledge base and various abilities. Setting up the curriculum and designing the questions according to the learning objectives are the key issues in PBL.

There are several inevitable limitations in this study. First, bibliometric data change with time, and different conclusions may be drawn with time; Second, the bibliometric analysis is only an auxiliary tool, and the results may differ from real-world research conditions; Third, the literature search was limited to Web of Science Core Collection databases, which might have resulted in an election bias to the outcomes; Forth, we limited the search term for the study topic to “PBL,” some relevant articles may be missed, such as “PBL.”

## Conclusion

The current study provides an overview of research findings and valuable insights into PBL worldwide. Research on PBL has continued to increase over the past few decades. The most productive country is the United States, participating in nearly 30% of publications, and the leading institution is the Maastricht University. The most attractive journal in terms of PBL is *Medical Education*. In addition, collaborative research initiatives need to be established between institutions in developing countries and those in developed countries.

## Data Availability Statement

The raw data supporting the conclusions of this article will be made available by the authors, without undue reservation.

## Author Contributions

FZ: conceptualization. FZ and HW: methodology and writing-original draft preparation. FZ and YB: software and data curation. HCZ: writing-review and editing. All authors have read and agreed to the published version of the manuscript.

## Conflict of Interest

The authors declare that the research was conducted in the absence of any commercial or financial relationships that could be construed as a potential conflict of interest.

## Publisher’s Note

All claims expressed in this article are solely those of the authors and do not necessarily represent those of their affiliated organizations, or those of the publisher, the editors and the reviewers. Any product that may be evaluated in this article, or claim that may be made by its manufacturer, is not guaranteed or endorsed by the publisher.
